# Management and long-term serum urate control in gout: a population-based primary care cohort study

**DOI:** 10.3399/BJGP.2025.0788

**Published:** 2026-07-14

**Authors:** Maria Antonia Pou, Carlen Reyes, Daniel Martinez-Laguna, Pau Satorra, Cristian Tebe, Nicola Dalbeth, Cesar Diaz-Torne

**Affiliations:** 1 Departament de Medicina, Universitat Autònoma de Barcelona, Catalonia, Spain; 2 Encants Primary Health Care Center, Institut Català de la Salut, Barcelona, Spain; 3 GREMPAL Research Group, IDIAP Jordi Gol, Barcelona, Barcelona, Catalonia, Spain; 4 Sardenya Primary Health Care Center, Barcelona, Spain; 5 Sant Marti de Provençals Primary Health Care Center, Institut Català de la Salut, Barcelona, Spain; 6 Germans Trias i Pujol Research Institute and Hospital (IGTP), Badalona, Spain; 7 Department of Medicine, University of Auckland, Auckland, New Zealand; 8 Rheumatology Department, Hospital de la Santa Creu i Sant Pau, Barcelona, Spain

**Keywords:** allopurinol, electronic medical record, febuxostat, gout, hyperuricaemia, primary health care

## Abstract

**Background:**

Gout is a prevalent chronic inflammatory disease associated with a substantial clinical and cardiovascular burden. Despite the availability of effective urate-lowering therapies (ULTs), gout management remains suboptimal.

**Aim:**

To describe clinical characteristics and treatment patterns, and to identify factors associated with long-term serum urate (SU) control in gout.

**Design and setting:**

Retrospective population-based cohort study using the primary care research database ‘Sistema d’Informació per al Desenvolupament de la Investigació en Atenció Primària’ (SIDIAP), covering >75% of the Catalan population within a universal healthcare system.

**Method:**

Individuals with incident gout diagnosed between 2012 and 2023 were included. Demographic, clinical, and ULT data were collected. Good SU control was defined as SU levels <6 mg/dL for ≥80% of follow-up time. Factors associated with SU control were analysed.

**Results:**

A total of 94 759 patients were included (mean age 66.25 [standard deviation 14.64] years; 79.58% male [75 412/94 759]; median follow-up 5.44 [interquartile range 2.54–8.24] years). In the 94 759 patients, hypertension (65.15%, *n* = 61 732), dyslipidaemia (50.58%, *n* = 47 932), diabetes (24.19%, *n* = 22 919), and chronic kidney disease (32.80%, 27 013/82 270 ) were common. Of the patients, 88.95% (*n* = 84 288) had ≥1 SU measurement. In total, 37.30% (35 343/94 759) had never received ULT. Allopurinol was the initial therapy in 96.09% of treated patients. Only 12.40% (10 453/84 288) achieved good SU control, with no improvement over time. Short ULT duration (≤25 days) occurred in 15.92% (9459/59 416) of patients. Good control was associated with longer treatment duration, higher allopurinol doses, febuxostat use, and better adherence, whereas delayed ULT initiation, male sex, obesity, chronic kidney disease, dyslipidaemia, and hypertension were associated with poor control.

**Conclusion:**

Long-term SU control in gout remains poor in primary care. Early initiation of ULT, appropriate dose optimisation, and improved adherence represent key modifiable targets to improve gout management.

**How this fits in**

Gout remains poorly controlled worldwide despite advances in understanding and treatment. It is associated with a high burden of cardiovascular comorbidities. In this cohort, serum urate control remained suboptimal and has not improved over the past decade. Male sex, obesity, chronic kidney disease, dyslipidaemia, and hypertension were associated with poor control of serum urate. Early urate-

lowering therapy initiation appears as a new modifiable factor that can improve the long-term care of people with gout.

## Introduction

Gout is the most common inflammatory arthropathy and its incidence continues to rise as a result of population ageing and the global obesity epidemic, both driven by lifestyle and dietary changes.^1^ Gout is associated with substantial comorbidity. Individuals with gout have an independent increased risk of cardiovascular disease and mortality,^2^ correlating with the extent of monosodium urate crystal deposition^3^ and a persistent proinflammatory state, even during asymptomatic periods.^4^ Gout arises from the chronic deposition of monosodium urate crystals and a greater crystal burden is linked to more severe articular and extra-articular manifestations. Importantly, these deposits may gradually diminish or even resolve if serum urate (SU) levels are maintained at a consistently low level over time, highlighting the need for sustained urate control.^1^

Multiple studies have shown that gout is often poorly managed across different settings. The causes are multifactorial, but several factors have consistently emerged in previous research: a low proportion of patients on urate-lowering therapy (ULT), inadequate dose escalation, and poor adherence.^5^ It is therefore important to understand why these patterns occur and to identify strategies, areas for improvement, and facilitators that may enhance management — particularly in primary care, where most patients with gout are followed up. This is not helped by the lack of consensus between guidelines, with the American College of Physicians^6^ recommending a more conservative approach to ULT initiation, while the European Alliance of Associations for Rheumatology (EULAR), National Institute for Health and Care Excellence (NICE), and American College of Rheumatology (ACR)^7–9^ support a treat-to-target strategy.

Previous studies have analysed gout characteristics and management, but many are cross-sectional,^10–13^ or include only treated patients, introducing potential bias towards good management or patients with severe gout.^14–17^

This study included a large cohort from 332 primary care centres across Catalonia, representative of the entire population of this region of Spain.^18^ To the authors’ knowledge, this is the first population-based study of this magnitude conducted in Spain and continental Europe. It includes both patients who were treated and those who were not, and allows analysis of management and control of SU over time. The study evaluates SU control not through cross-sectional measurements, but continuously and longitudinally, providing insight into treatment patterns and disease management over time, which is the most appropriate approach given the chronic nature of the disease. The objective was to characterise a cohort with incident gout diagnosed between 2012 and 2023, including sociodemographic and clinical characteristics, cardiovascular comorbidities, drug utilisation, treatment patterns, and adherence to ULT, and analyse factors associated with SU control.

## Method

### Data source

Data from the primary care research database ‘Sistema d’Informació per al Desenvolupament de la Investigació en Atenció Primària’ (SIDIAP) was used,^18^ which contains coded information in routine primary care records, covering >75% of the Catalan population (6 million individuals). The database includes demographic and clinical information (International Classification of Diseases-10 Clinical Modification codes), prescriptions, and dispensations from community pharmacy claims (Anatomical Therapeutic Chemical [ATC] codes). The validity of gout diagnoses in SIDIAP has been confirmed.^19^

In Spain, health care is universal and free of charge. Each citizen is assigned a family doctor who records clinical information in the electronic health record system, which serves as the main data source for SIDIAP.

### Study design and participants

A retrospective, population-based cohort study was conducted including patients ≥15 years with an incident gout diagnosis between 2012 and 2023. Patients were followed until death, transfer out of the region, or the administrative end of the study (31 December 2023), whichever occurred first, and follow-up was censored accordingly. Routine monitoring of patients with chronic diseases minimises the risk of prolonged periods without encounters.

Patients were excluded if they had no clinical data in the 12 months before inclusion or had a diagnosis of another inflammatory arthropathy (rheumatoid arthritis, psoriatic arthritis, or chondrocalcinosis).

### Data collection

Baseline characteristics included age, sex, diagnosis date, deprivation index (MEDEA Index: this is divided into quintiles, with the higher numbers representing the more severe levels of deprivation. Employment and education are the dominant domains),^20^ alcohol abuse (men ≥28 standard drink units/week; women ≥17 standard drink units/week), SU, estimated glomerular filtration rate (eGFR), and body mass index (BMI).

Baseline comorbidities included hypertension, dyslipidaemia, type 2 diabetes, ischaemic heart disease (IHD), obesity, cerebrovascular disease (CVD), chronic kidney disease (CKD), and the Charlson Comorbidity Index.

ULT data included drug (allopurinol, febuxostat, benzbromarone), dose, and duration. Treatment periods were defined by consecutive dispensations of the same drug separated by <90 days. The mean prescribed dose per patient and drug was used as a proxy for treatment dose. The full list of codes used to define variables is provided in Supplementary Information S1. Only 24 (0.04%) individuals received benzbromarone; because of this limited sample they were excluded from comparisons. Benzbromarone is rarely used in Spain, reserved for patients with severe gout unresponsive to allopurinol or febuxostat, and its prescription is restricted to rheumatologists or nephrologists (Supplementary Information S2).

### Outcomes

The primary outcomes were SU control and ULT adherence. SU target was defined as an SU level <6 mg/dL (360 µmol/L).^7^ To incorporate time, SU control was categorised into four groups based on the proportion of time the target was achieved: null control (never), poor control (<50%), moderate control (50–80%), and good control (≥80%). Control time was interpolated between consecutive measurements: half the interval was assigned to the earlier SU, half to the later. Before the first measurement, half of the period was assumed uncontrolled; after the last, only the first half of the interval was used to avoid overestimation of control time.

Adherence was measured by the Medication Possession Ratio (MPR), reflecting the percentage of time patients had gaps (<90 days without treatment) inside a treatment period. Good adherence was defined as MPR ≥80%. Patients with a single dispensation were excluded, as at least two are required to estimate adherence over time.

### Statistical analysis

Population characteristics were described with mean and standard deviation (SD), median and interquartile range (IQR), or proportion. ULT initiation and prevalence were calculated overall and by year, stratified by sex. ULT annual prevalence was defined as having an active ULT dispensation in January among all patients in the study period in that year. ULT annual initiation was defined as having a new ULT dispensation at any point during the year, among patients without an active dispensation in January or in the preceding 3 months.

Initiation and prevalence of SU control (<6 mg/dL; 360 µmol/L) were assessed annually and overall, stratified by sex. Good control annual prevalence was defined as having good control on 1 January among all patients in the study period in that year. Good-control annual initiation was defined as achieving good control at any point of the year, among patients without good control on 1 January.

Good adherence rates were calculated as the proportion of treated patients in each year who had good adherence.

Logistic regression models were used to analyse the raw association of a set of factors with good SU control for exploratory purposes. The study also analysed the association of good adherence and time until first treatment with SU control using logistic regression, both raw and adjusted by age, sex, eGFR, type 2 diabetes, and hypertension. Odds ratios (ORs) are reported. Statistical analyses were performed using R software version 4.3.0 or higher. Statistical significance was set at 5%. Model assumptions were assessed, and estimators were reported with 95% confidence intervals.

### Patient and public involvement

Patients with gout helped design the study and identify key outcomes, noting issues such as infrequent ULT initiation, titration, and urate monitoring, as well as differences in disease management among healthcare providers. They, together with clinicians and the public, will be invited to future engagement events to review findings and guide follow-up projects aimed at improving care.

## Results

### Study population and baseline characteristics

**Figure 1.**
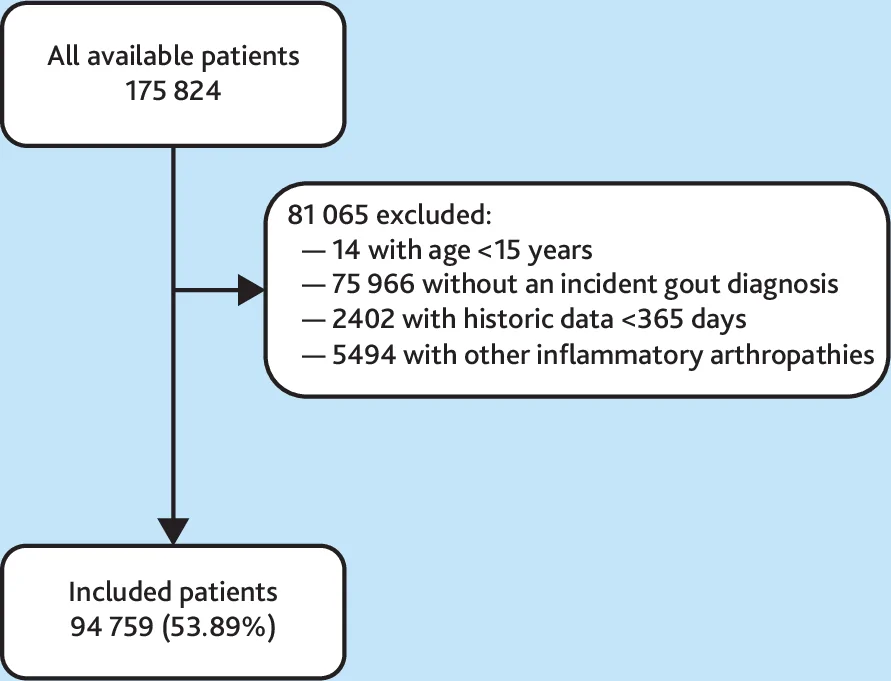
Flowchart of the study.^a a^Some exclusions fit in more than two categories.

A total of 94 759 individuals with incident gout were included between January 2012 and December 2023, contributing 519 910 person-years of follow-up (Figure 1).

During follow-up, 18 353 (19.36%) died and 2944 (3.11%) were transferred to another health region. Median follow-up was 5.44 years (IQR 2.54–8.24). Incident gout diagnoses included per year are shown in Supplementary Table S1.

Baseline characteristics are summarised in Table 1. The most common comorbidities were hypertension (65.15%, 61 732/94 759) and dyslipidaemia (50.58%, 47 932/94 759). Mean BMI was 30.10 kg/m² (SD 5.02).

**Table 1.** Baseline characteristics of the cohort (*N* = 94 759)

**Characteristic**	**Patients**	**Total *N***
**Sex, male**	75 412 (79.58)	94 759
**Age, years, mean (SD)**	66.25 (14.64)	94 759
**Area**		77 785
Urban	58 459 (75.15)	
Rural	19 326 (24.85)	
**Socioeconomic status using MEDEA index** ^**a**^		58 459
U1	10 645 (18.21)	
U2	11 574 (19.80)	
U3	12 598 (21.55)	
U4	12 167 (20.81)	
U5	11 475 (19.63)	
**Serum urate,** ^**b**^ **mg/dL, median (IQR)**	7.90 (6.60–8.90)	81 940
**BMI category**		64 310
Underweight: <18.5 kg/m^2^	187 (0.29)	
Normal weight: 18.5–24.9 kg/m^2^	8217 (12.78)	
Overweight: 25.0–29.9 kg/m^2^	26 393 (41.04)	
Obesity class I: 30.0–34.9 kg/m^2^	19 865 (30.89)	
Obesity class II: 35.0–39.9 kg/m^2^	7062 (10.98)	
Obesity class III: ≥40.0 kg/m^2^	2586 (4.02)	
**CKD (eGFR), mL/min/1.73m** ^**2**^		82 270
I–II	55 257 (67.17)	
IIIa	14 224 (17.29)	
IIIb	9189 (11.17)	
IV	3261 (3.96)	
V	339 (0.41)	
**Dyslipidaemia**	47 932 (50.58)	94 759
**Hypertension**	61 732 (65.15)	94 759
**Type 2 diabetes mellitus**	22 919 (24.19)	94 759
**Ischaemic heart disease**	10 426 (11.00)	94 759
**Cerebrovascular disease**	6364 (6.72)	94 759
**Alcohol abuse** ^**c**^	2194 (4.57)	48 032
**Charlson Index, median (IQR)**	3.0 (1.0–4.0)	14 903

Data are *n* (%) unless otherwise specified. ^a^MEDEA Index: U1 less deprived; U5 most deprived. ^b^Measured before or at gout diagnosis. ^c^Alcohol abuse: men ≥28 standard drink units/week; women ≥17 standard drink units/week. CKD = chronic kidney disease. eGFR = estimated glomerular filtration rate. IQR = interquartile range. SD = standard deviation.

### ULT characteristics

Of the 94 759 patients there were 35 343 (37.30%) people who never received ULT. Treated patients were on ULT for a median of 29.44% (IQR 9.36– 51.41) of the time. Median time from diagnosis to first ULT was 56.00 days (IQR 16.00–263.14). Median duration of the first ULT was 114.69 days (IQR 24.00–299.00). There were 15.92% (9459/59 416) that received a single dispensation every time they began ULT. The patients’ characteristics according to allopurinol or febuxostat last treatment and non-treated are shown in Table 2.

**Table 2.** Characteristics according to the type of treatment received^a^ (allopurinol or febuxostat) and patients who did not receive treatment

Characteristic	Allopurinol	Febuxostat	No urate-lowering therapy
**Number of patients** ^**b**^	55 290 (58.35)	4102 (4.33)	35 343 (37.30)
**First-line treatment**	54 120 (96.09)	2202 (3.91)	
**Sex, male**	44 889 (81.19)	3285 (80.08)	27 220 (77.02)
**Age, years, median (IQR)**	72.37 (61.70–81.70)	70.71 (60.04–80.21)	70.42 (57.75–81.33)
**MPR %, median (IQR)**	44.87 (37.32–65.59)	85.97 (66.35–94.65)	
**MPR ≥80%**	2027 (6.01)	1750 (58.41)	
**Days of treatment, median (IQR)**	116.00 (25.00–299.00)	299.50 (59.00–850.00)	
**BMI category**	38 237	2736	16 251
Underweight: <18.5 kg/m^2^	132 (0.35)	12 (0.44)	157 (0.97)
Normal weight: 18.5–24.9 kg/m^2^	5515 (14.42)	384 (14.04)	3275 (20.15)
Overweight: 25.0–29.9 kg/m^2^	15 344 (40.13)	1078 (39.40)	6682 (41.12)
Obesity class I: 30.0–34.9 kg/m^2^	11 418 (29.86)	810 (29.61)	4264 (26.24)
Obesity class II: 35.0–39.9 kg/m^2^	4229 (11.06)	327 (11.95)	1347 (8.29)
Obesity class III: ≥40.0 kg/m^2^	1599 (4.18)	125 (4.57)	526 (3.24)
**CKD (eGFR), mL/mn/1.73m** ^**2**^	47 938	3549	21 994
I–II	27 788 (57.96)	1525 (42.97)	14 433 (65.62)
IIIa	9250 (19.30)	705 (19.86)	3510 (15.96)
IIIb	6915 (14.43)	800 (22.54)	2451 (11.14)
IV	3385 (7.06)	431 (12.14)	1252 (5.69)
V	600 (1.25)	88 (2.48)	348 (1.58)
**Dyslipidaemia**	32 081 (58.02)	2377 (56.95)	18 169 (51.41)
**Hypertension**	41 746 (75.50)	3165 (77.16)	21 855 (61.84)
**Type 2 diabetes mellitus**	17 015 (30.77)	1242 (30.28)	9531 (26.97)
**Ischaemic heart disease**	7926 (14.34)	503 (12.26)	4378 (12.39)
**Cerebrovascular disease**	5172 (9.35)	367 (8.95)	3391 (9.59)
**Alcohol abuse** ^**c**^	1080 (3.61)	58 (2.79)	536 (3.27)

Data are *n* (%) unless otherwise indicated. The percentages of number of patients and first-line treatment are based on row total *N.* MPR *≥*80% cannot be calculated in patients with single dispensations. Totals are: allopurinol 2027/33 752 and febuxostat 1750/2996.Total N for alcohol abuse are: allopurinol 1080/29 897; febuxostat 58/2080; non-ULT: 536/16 386. Patient characteristics were taken at the start of the dominant treatment. There were just 24 (0.04%) individuals finally treated with benzbromarone. Alcohol abuse: men ≥28 standard drink units/week; women ≥17 standard drink units/week. BMI = body mass index. CKD = chronic kidney disease. eGFR = estimated glomerular filtration rate. MPR = medication possession ratio. ULT = urate-lowering therapy.

Treated patients generally exhibited a higher comorbidity burden than untreated. Allopurinol was the first-line treatment in 96.09% (54 120/56 322). Of these, 3.49% (1889/54 120) switched to febuxostat. Overall, febuxostat was more commonly used in patients with CKD (57.03% [2024/3549] versus 42.04% [20 150/47 928]). Adherence was also superior among febuxostat users. Febuxostat was generally used in patients with higher baseline SU (Supplementary Table S2).

**Figure 2.**
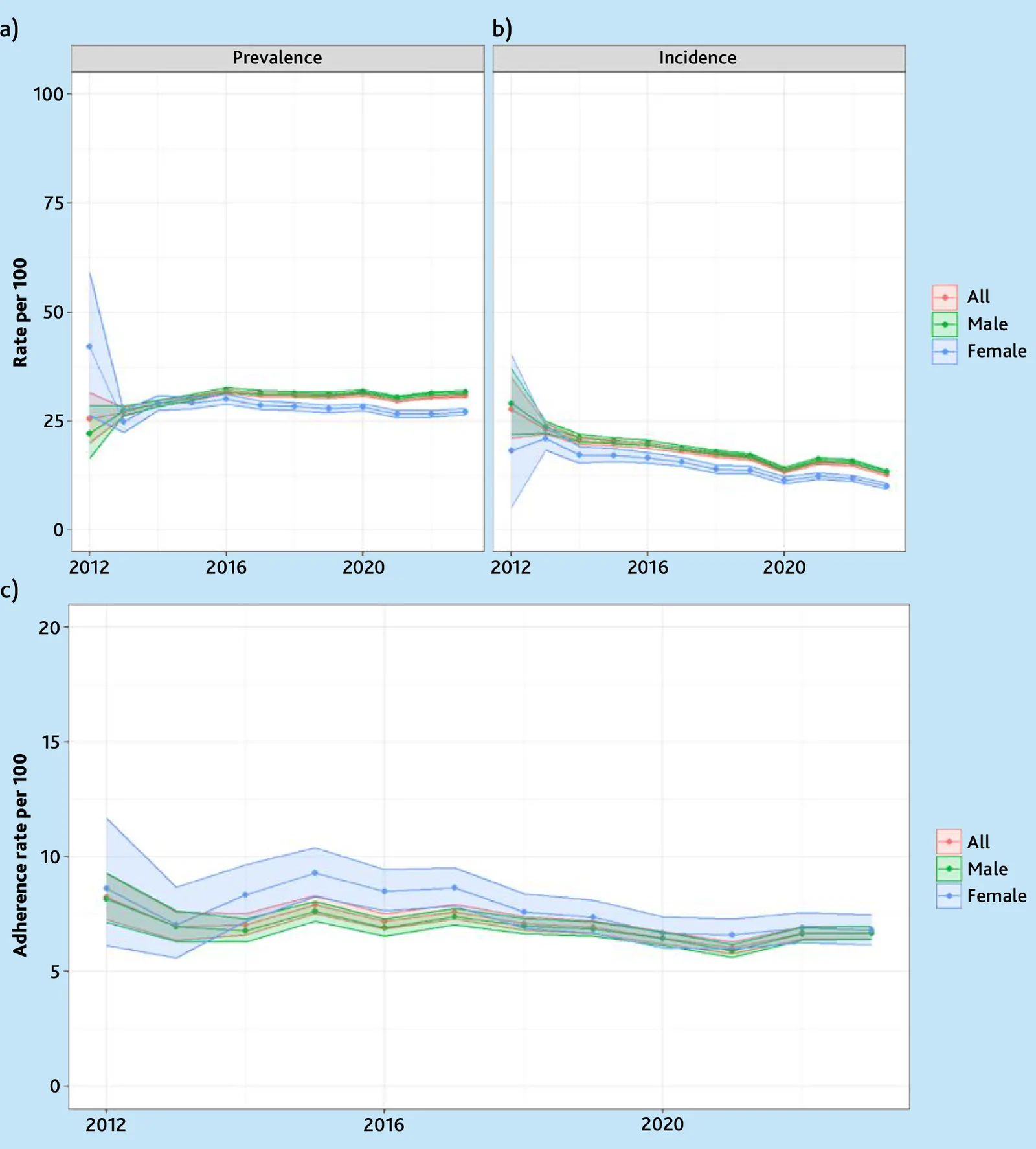
a) Prevalence and b) incidence of ULT throughout the follow-up period. c) Good adherence (MPR ≥80%) to ULT rate. MPR = Medication Possession Ratio. ULT = urate-lowering therapy.

Overall, 59 416 of 94 759 individuals (62.70%) were dispensed ULT. As shown in Figure 2, the prevalence of ULT remains stable throughout the cohort’s follow-up, whereas its initiation and good adherence show a slow and progressive decline. For all, treatment prevalence ranged from 25.34% (year 2012) to 31.74% (year 2016), while initiation declined from 27.88% (year 2012) to 12.59% (year 2023). Good adherence (MPR ≥80%) was achieved by only 6.06% (1969/32 830) (year 2021) to 8.07% (236/2294) (year 2012) of all patients. Although males were more frequently treated, females demonstrated better adherence.

#### SU control

**Figure 3.**
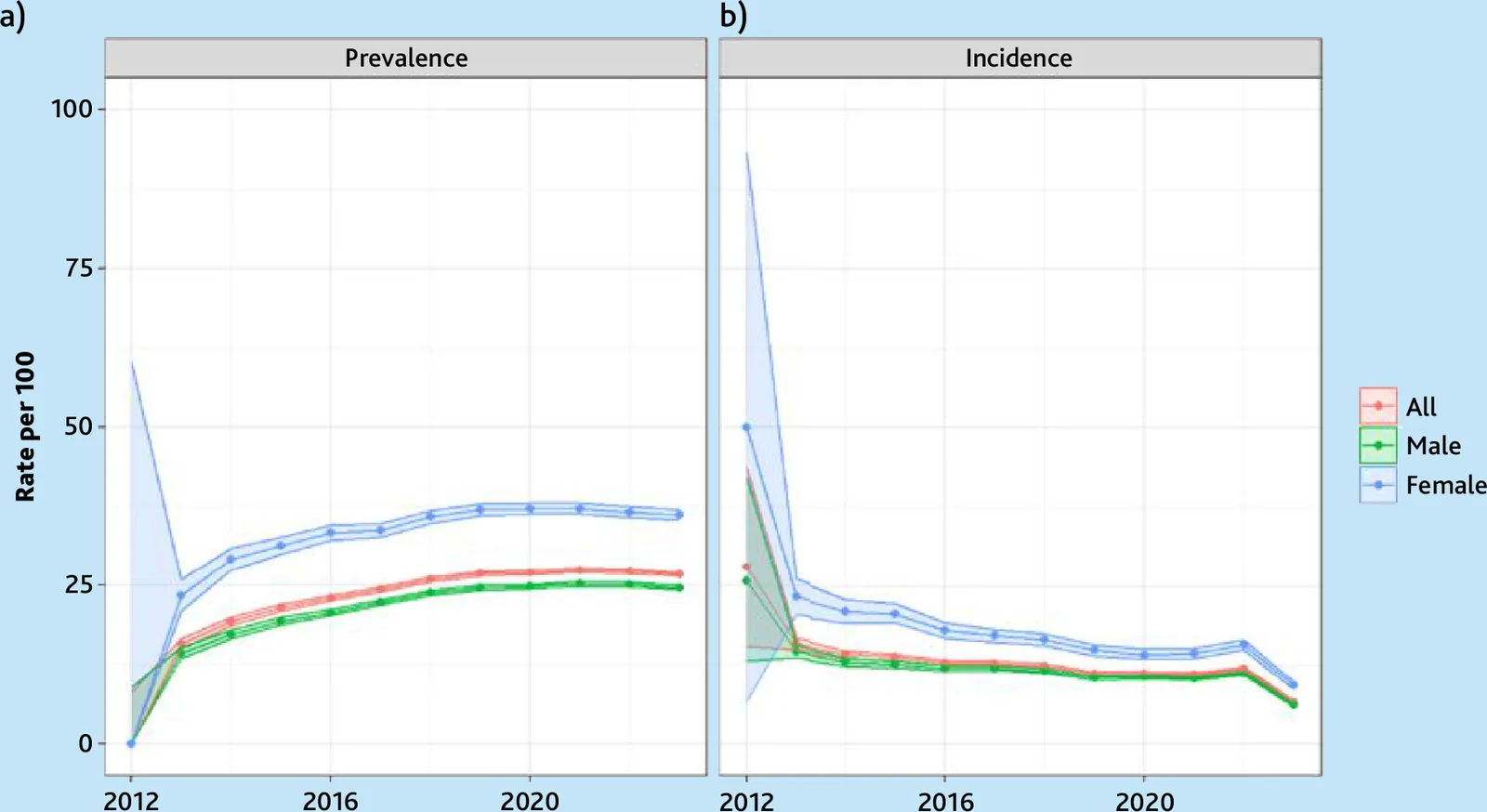
a) Prevalence and b) incidence of SU good control (≥80% of time SU <6 mg/dL) throughout the follow-up period. SU = serum urate.

SU levels were measured in 88.95% (84 288/94 759) of the patients. Good SU control was achieved in 10 453 (12.40%), while 11 137 (13.21%) had moderate control, 23 380 (27.74%) poor control, and 39 318 (46.65%) null control. The prevalence of good SU control increases slowly and progressively throughout the study period, whereas the incidence shows a minimal decline (Figure 3).

Among the 53.35% (44 970/84 288) of the individuals who achieved an SU level <6 mg/dL at least once during follow-up, SU remained below this threshold for a median of 47.77% (IQR 23.06–77.63) of their total follow-up time. The proportion of patients with moderate or good control did not vary much by the number of SU tests, duration of follow-up, or year of diagnosis (Supplementary Tables S3–S5).

**Figure 4.**
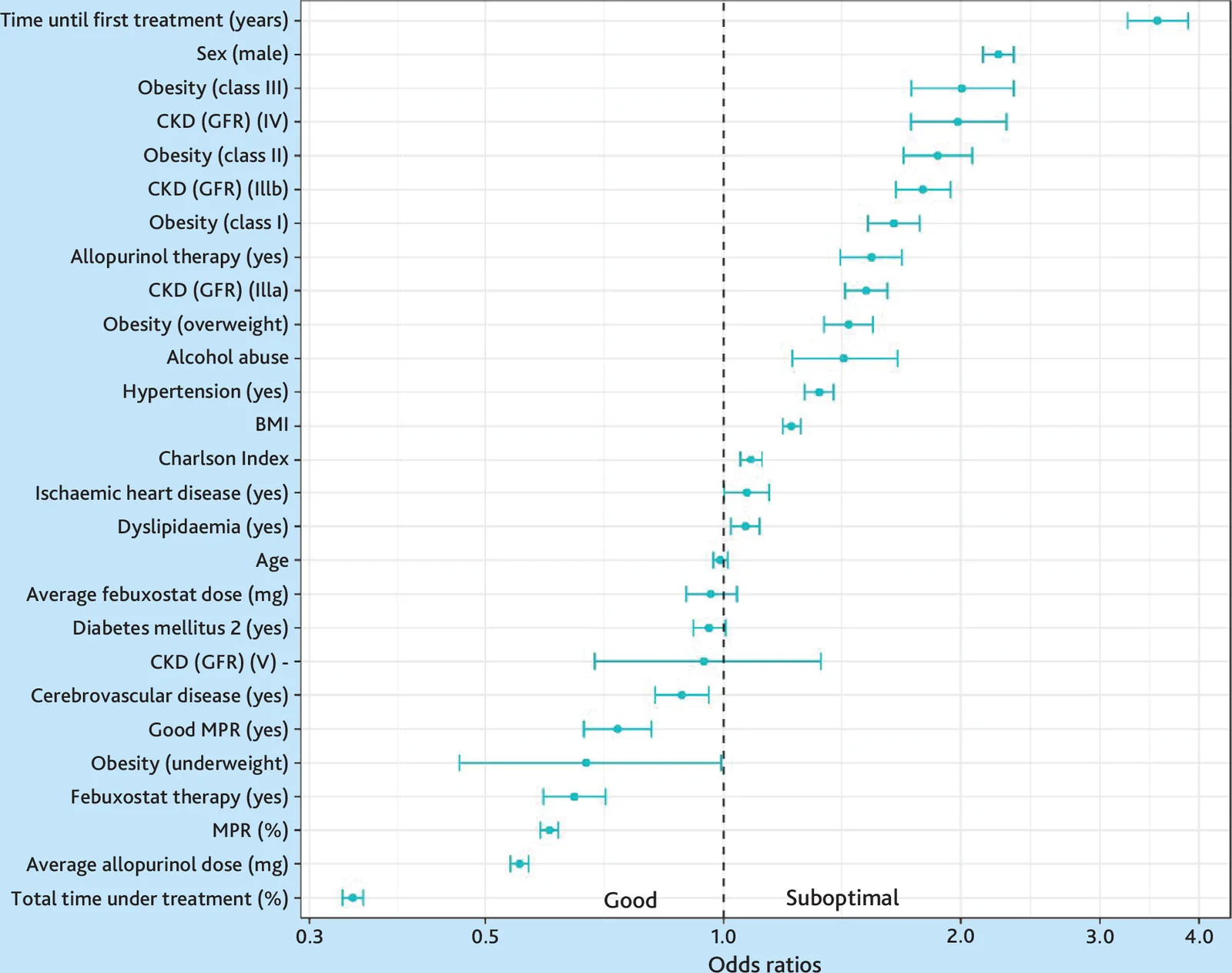
Clinical and dominant treatment factors associated with suboptimal serum urate control. BMI = body mass index. CKD = chronic kidney disease. GFR = glomerular filtration rate. MPR = medication possession ratio.

Delayed treatment initiation, allopurinol therapy, male sex, higher BMI, obesity, hypertension, dyslipidaemia, CKD, IHD, higher Charlson index, and alcohol abuse were associated with poorer control. Conversely, CVD, type 2 diabetes, longer proportion of time under treatment, higher allopurinol doses, febuxostat therapy, and better adherence correlated with improved control (Figure 4 and Supplementary Table S6). The median allopurinol dose among patients in the well-controlled group was 212.5 (IQR 100–300) mg/day, higher than in moderate (150.0, IQR 100–275), poor (108.3, IQR 100–200), or null control groups (100.0, IQR 100–150).

The association between adherence and proportion of time under treatment with good control is represented in Supplementary Figure S1. The values for adherence and proportion of time under treatment increased for categories with better control.

In the multivariable models, better adherence (OR 95% CI = 1.73, 1.68–1.77) was associated with improved SU control while more time until treatment initiation (OR 95% CI = 0.28, 0.25–0.31) was associated with worse SU control (Supplementary Table S7). When analysing the relationship between baseline SU levels and the achievement of good control, it was observed that it was more frequent among patients with levels <7 mg/dL, whereas above this threshold no differences were found across the different SU levels (Supplementary Table S8).

## Discussion

### Summary

Data from a large, population-based cohort of 94 759 patients with incident gout were analysed, followed for >10 years in a universal healthcare setting. The study included patients whose condition was treated and those where it was untreated and allowed a longitudinal evaluation of SU and made it possible to identify factors associated with optimal management. Comorbidities were highly prevalent (hypertension 65.15% [61 732/94 759], dyslipidaemia 50.58% [47 932/94 759], type 2 diabetes 24.19% [22 919/94 759]), and 62.70% (59 416/94 759) of patients received ULT. Overall, SU control was poor, with <13% achieving sustained target levels. Better control was associated with early ULT initiation, higher allopurinol doses, febuxostat, longer treatment duration, and better adherence, whereas male sex, obesity, alcohol abuse, and CKD were linked to poorer control.

### Strengths and limitations

One of the main strengths of this study is that it represents, to the authors’ knowledge, one of the largest population-based cohorts of patients with gout to date, integrating clinical, laboratory, and dispensing data from primary care, where over 90% of patients are managed. The SIDIAP database ensures broad representativeness (covering >75% of the population) and data completeness.^21^ Another strength of the current study is the longitudinal analysis of SU control — unlike prior cross-sectional reports^11,13,14,17,22^ — that provides a realistic assessment of disease control over time thus recognising gout as a chronic disease, and linkage to pharmacy-dispensing data (ATC codes), allowing reliable evaluation of ULT adherence and dosing. Unlike most studies, patients who were not treated with ULT were not excluded, avoiding selection bias and improving real-world validity. Another strength is that both single and multivariable analyses were conducted to evaluate factors associated with good SU control, and the results were very similar, which reinforces the robustness of these findings.

As with all electronical medical record-based studies, misclassification of gout diagnosis is possible, although prior validation supports data reliability.^19^ Large primary care databases may also not capture the nuances of individual treatment decisions. Other limitations include lack of data on gout flares, quality of life, use of diuretics or other drugs that may influence SU levels, and mortality outcomes. Another limitation is the absence of ethnicity data, as Spanish legislation does not permit their routine collection in health records. Although total follow-up spanned 12 years, the median follow-up (5 years) may still underestimate long-term management dynamics. Nonetheless, this time exceeds that of most published cohorts.

### Comparison with existing literature

The current cohort’s demographic and clinical characteristics are consistent with previous studies from the Mediterranean region.^22–24^ Compared with non-European cohorts, variability emerges: US studies report similar or higher comorbidity and obesity rates,^12,14,25^ whereas Asian cohorts describe younger populations with less obesity but with persistent cardiometabolic comorbidities.^11,26,27^ Differences likely reflect healthcare structures, regional patterns, and study designs, as some previous cohorts included only patients who received treatment or specific subpopulations (such as Veterans Affairs in the US, or treated-only cohorts in the UK).^16,28^ The data from this study and the published previously one reinforce that cardiovascular comorbidities are central to gout management and should guide individualised, comorbidity-focused strategies.^29^

Only 62.70% (59 416/94 759) of patients in the current cohort received ULT, and adherence was poor. Similar underutilisation has been reported elsewhere, with treatment rates ranging from 27% to 66% in other population-based cohorts,^12,30^ and 47% in Veterans Affairs patients.^31^ These rates have not improved substantially in the past decade.^32^ In Spain, as in most other countries, gout management remains outside chronic disease protocols and most family physicians report limited familiarity with guideline recommendations.^33^ Moreover, 15.92% (9459/59 416) of patients had a single ULT dispensation, suggesting persistent misperceptions of gout as an acute condition rather than a chronic disease.^34^ Differences in guideline recommendations may contribute to this variability. The American College of Physicians (ACP) advises against starting ULT after a first flare or infrequent flares,^6^ whereas ACR, EULAR, the Spanish clinical practice guideline, and NICE recommend a treat-to-target strategy.^7–9,35^ The ACP position, however, reflected the limited long-term evidence available then, rather than evidence against ULT efficacy.

The proportion of patients achieving SU <6 mg/dL (360 µmol/L) was markedly low and stable over time, consistent with prior evidence showing suboptimal control globally.^17,23,36^ The proportion in the current study (<13% with good control) is comparable with, or slightly lower than, other real-world studies, highlighting that long-term control remains inadequate despite guideline recommendations. Another important finding is the lack of progress in both ULT initiation and achievement of the therapeutic target. Although some positive changes have been reported in countries with similar healthcare systems, these remain largely insufficient.^13^ Unlike in some countries, SU control in the current cohort was not influenced by diagnosis year, follow-up duration, or socioeconomic status, aligning with results from universal-access health systems.^17,36^

The current results align with previous studies identifying pharmacological factors such as higher allopurinol doses, febuxostat use, treatment duration, and adherence as key determinants of control.^11,14,17^ In the current cohort, febuxostat use may be associated with better control, not only because allopurinol is often prescribed at low doses, but also because of higher adherence. The authors of the current study hypothesised that this improved adherence with febuxostat could be related to the fact that it may be prescribed by physicians with a greater interest in gout and to its use as a second-line therapy in patients with more severe disease, who may therefore be more aware of, and engaged in, their treatment.

Non-pharmacological predictors of poor control — male sex, obesity, alcohol abuse, hypertension, and CKD — were also consistent with the literature.^11,14,17,36^ Interestingly, CKD stage V was not associated with poorer control, possibly because of dialysis or renal transplant bias.^37^ In contrast to the STOP GOUT trial,^38^ the current study found that comorbidities influenced SU control, likely reflecting differences between real-world primary care populations and tightly managed clinical trial cohorts. The multivariable analysis of pharmacological factors reinforces the findings of the univariable analysis. In the current study the multivariable model was restricted to adherence and early treatment initiation because these are modifiable factors and relevant to clinical practice. The remaining variables (age, sex, and comorbidities) act as non-modifiable confounders, and their effects were already demonstrated in the univariable analysis. Early initiation of ULT was associated with improved SU control, reinforcing the need to complement the ‘start-low, go-slow’ ULT therapy principle^39^ with ‘and begin soon’. Although it is plausible that patients who start treatment earlier achieve better control, in the current study the difference in initiation time between the good-control and suboptimal control groups was relatively small (25.57 versus 69.40 days; a difference of 43.83 days). Given that such a modest temporal difference was associated with more than a threefold increase in the likelihood of achieving the target outcome, this suggests that additional, unmeasured factors — such as patient or physician attitudes, treatment expectations, or clinical decision-making patterns — may play an important role, as also illustrated by qualitative studies and expert opinion.^40–42^

Additionally, recent evidence indicates that initiating ULT during a flare does not worsen the duration or intensity of the flare.^43^ This raises the question of whether the optimal timing for ULT initiation might be during the flare itself. The authors of the current study believe that this approach could help patients better understand their disease by associating clinical improvement of the flare with ULT, while also aligning the prescription pattern more closely with that of other chronic diseases. Some of the similarities observed when comparing the current results with those from other countries with universal healthcare coverage reinforce the current findings and suggest that a strategy of early initiation of ULT may be particularly effective in this context.^13,17^

### Implications for practice

In conclusion, these findings suggest that long-term SU control in gout is less influenced by external socioeconomic factors or healthcare system accessibility than by treatment-related aspects. No relevant differences according to socioeconomic status, follow-up duration, or frequency of laboratory testing were observed, pointing instead to undertreatment and therapeutic inertia as key contributors to suboptimal control. Pharmacological management therefore remains a central area for improvement. Notably, early initiation of ULT was associated with better long-term urate control, suggesting that earlier treatment may be beneficial in routine clinical practice, especially in primary care.

## Data

The data supporting the findings of this study are not publicly available because of ethical and data protection restrictions. However, they may be made available from the corresponding author upon reasonable request and subject to the necessary approvals and regulatory requirements.
